# The impact of patient-reported visual disturbance on dynamic visual acuity in myopic patients after corneal refractive surgery

**DOI:** 10.3389/fnins.2023.1278626

**Published:** 2023-10-10

**Authors:** Yuexin Wang, Yu Zhang, Tingyi Wu, Xiaotong Ren, Yifei Yuan, Xuemin Li, Yueguo Chen

**Affiliations:** ^1^Department of Ophthalmology, Peking University Third Hospital, Beijing, China; ^2^Beijing Key Laboratory of Restoration of Damaged Ocular Nerve, Beijing, China

**Keywords:** visual disturbance, dynamic visual acuity, myopia, corneal refractive surgery, visual function

## Abstract

**Purpose:**

To investigate the impact of patient-reported visual disturbance on dynamic visual acuity in myopic patients after corneal refractive surgery.

**Methods:**

This is a prospective nonrandomized study. Adult myopic patients receiving bilateral photorefractive keratectomy (PRK), femtosecond laser-assisted *in situ* keratomileusis (FS-LASIK), or small incision lenticule extraction (SMILE) with Plano target were included. Eight types of patient-reported visual disturbance were evaluated regarding frequency, severity and bothersome and dynamic visual acuity (DVA) of 40 and 80 degrees per second (dps) was measured postoperatively at 3 months.

**Results:**

The study enrolled 95 patients with an average age of 27.6 ± 6.4 years. The most frequently reported visual disturbance was the fluctuation in vision (70.5%), followed by glare (66.3%) and halo (57.4%). Postoperative DVA at 80 dps was significantly associated with the total score of haloes (*p* = 0.038) and difficulty in judging distance (*p* = 0.046). Significant worse postoperative DVA at 40 dps was observed in patients with haloes than those without (*p* = 0.024). The DVA at 80 dps for patients without haloes or difficulty in judging distance was significantly better than that with the symptoms (haloes, *p* = 0.047; difficulty in judging distance, *p* = 0.029). Subgroup analysis by surgical procedures demonstrated that the significant difference in DVA between patients with and without visual disturbance was only observed in patients receiving FS-LASIK.

**Conclusion:**

Postoperatively, myopic patients undergoing corneal refractive surgery with haloes or difficulty in judging distance have significantly worse low and high-speed DVA than those without the symptoms. The present study provided the basis for postoperative guidance in daily tasks involving dynamic vision when patients have visual disturbances.

## 1. Introduction

Myopia is the leading cause of reversible visual impairment globally (Lou et al., 2016), and corneal refractive surgery has become an effective and frequently used method in myopia correction (Kim et al., 2019). There are mainly three types of surgeries, including corneal surface ablation techniques, corneal stromal ablation surgery and corneal lenticule extraction procedure (Wen et al., 2017). Extending beyond spectacle independence, the surgeries are considered to improve quality of life and daily working performance (Sugar et al., 2017). Thus, vision-related quality of life evaluation is crucial to assess the safety and effectiveness of the surgeries.

Traditional clinical examination of visual quality mainly focuses on static vision, including static visual acuity, contrast sensitivity, and wavefront aberration (Miao et al., 2017). Dynamic visual acuity (DVA) refers to the capacity to identify the detail of objects with relative motion, which is essential for daily tasks (Hirano et al., 2017; Wu et al., 2021). Examining visual acuity with moving optotypes could potentially better reflect real-life visual function. Thus, DVA is increasingly applied as an indicator to assess driving (Lacherez et al., 2014) and sports performance (Uchida et al., 2012) and vision-related quality of life in ocular disease (Ren et al., 2020; Wu et al., 2021). Previous research demonstrated that myopic patients achieved improved DVA after corneal refractive surgery than preoperative measurements with corrected spectacles (Wang et al., 2023a,b).

In addition to clinical vision-related examinations, patient-reported outcome questionnaires have been an increasingly popular method to assess subjective visual perception and disturbance after refractive surgery (Schallhorn et al., 2016; Sugar et al., 2017; Schmelter et al., 2019; Siedlecki et al., 2020; He et al., 2022). The questionnaire evaluates visual disturbance frequently reported after corneal refractive surgery, including glare, halo, starburst, and so on, despite 20/20 static visual acuity (Schmelter et al., 2019; Siedlecki et al., 2020; He et al., 2022). These patient-reported outcomes could better indicate the effect of refractive surgery on the patient’s visual performance in daily tasks. Thus, the frequency, severity and bothersome of the visual disturbance might influence the ability of the subjective to identify moving objects. At present, however, the evidence on the impact of subjective visual disturbance on DVA is limited.

The present research aims to investigate the impact of visual disturbance on DVA in myopic patients after corneal refractive surgery using commonly applied, validated patient-reported quality of vision questionnaires. The study helps us to understand the subjective influential factor on DVA and might guide dynamic vision-related daily tasks in patients with visual disturbance after corneal refractive surgery.

## 2. Materials and methods

### 2.1. Participants

The present research was a prospective, non-randomized case series enrolled patients undergoing corneal refractive surgery. The study was conducted following the tenets of the Declaration of Helsinki, and the research protocol was approved by the local review board (M2020431). Informed consent was obtained from each subject.

Consecutive patients undergoing bilateral photorefractive keratectomy (PRK), femtosecond laser-assisted *in situ* keratomileusis (FS-LASIK) or small incision lenticule extraction (SMILE) were prospectively enrolled. The inclusion criteria were as follow: (Lou et al., 2016) age 18–40 years of age (Kim et al., 2019), correction of myopia or myopic astigmatism for Plano target; (Wen et al., 2017) pre-operative and three-month postoperative corrected distance visual acuity (CDVA) 0 (LogMAR) or better. The exclusion criteria were: (Lou et al., 2016) history of severe ocular diseases, including glaucoma, retinal disease, or severe ocular surface disease; (Kim et al., 2019) vestibular dysfunction or cognitive disorder that affects the DVA test.

### 2.2. Preoperative and postoperative static vision evaluation

All enrolled patients underwent comprehensive preoperative evaluation, including uncorrected distance visual acuity (UDVA, LogMAR visual chart), cycloplegic and non-cycloplegic subjective refraction with CDVA, slit-lamp biomicroscope, fundoscopy and corneal topography (Pentacam, Oculus, Germany). All patients were required to be examined at 1 week, 1 month and 3 months postoperatively. UDVA, non-cycloplegic subjective refraction with CDVA and slit-lamp biomicroscope were examined at one and 3 months postoperatively.

### 2.3. Dynamic visual acuity test

The DVA was evaluated with the previously reported test system (Wang et al., 2023a,b). We assess binocular DVA at 40 and 80 degrees per second (dps) with the naked eye at 3 months postoperatively. The test was arranged at a 2.5 m distance in a quiet room with 30 lux overhead illumination, and the optotypes were presented on a 14-inch 120 Hz screen. The test software was programmed with MATLAB 2017b (MathWorks, United States), which could demonstrate the horizontal moving letter E with a certain size and speed. The optotype E was designed according to the LogMAR visual chart, and the moving speed was quantified as the changing view angle per second.

During the test, the optotypes appeared in the middle of the screen’s left side and horizontally moved to the middle of the right side. The patient was required to identify the opening direction of the letter E during the movement. The initial optotype was three sizes bigger than the UDVA. For each size, we presented eight optotypes one by one with a random opening direction. The optotype would be switched to one size smaller if the patient could identify five out of eight optotypes correctly. We recorded the minimum size (A, logMAR) the participant could recognize (no less than five out of eight) and the number (b) of identified optotypes one size smaller, and the result was calculated with the previously reported formula as follows (Wang et al., 2023a):


$$DVA=A-\frac{0.1}{8}*b$$

### 2.4. Surgical procedures.

The surgical procedures were selected according to the corrected distance VA, refraction, corneal topography, aberration and patients’ intention following sufficient informed consent. Before the surgery, the eyelids were prepared with a 5% povidone-iodine solution, and oxybuprocaine hydrochloride was applied for topical anesthesia. The surgical procedures were as follows:

PRK: The epithelial was removed after soaking in 20% ethanol for 20 s. The ablation was conducted with a Custom Q profile by WaveLight EX500 excimer laser (Alcon Laboratories Inc., Fort Worth, United States). After ablation, a 0.02% mitomycin C pad was covered on the residual stroma. Then the stroma was rinsed with normal saline. A bandage contact lens (Acuvue, Johnson Vision Care. Inc., United States) was placed on the cornea for protection.

FS-LASIK: A elliptical flap was created with WaveLight FS200 laser (Alcon Laboratories Inc., Fort Worth, United States) with a thickness of 110 mm and a diameter of 8.5 to 9.0 mm. The flap was lifted, and the ablation was performed with Topography guided profile by WaveLight EX500 excimer laser. Afterward, the flap was repositioned properly without striae.

SMILE: The intrastromal lenticule was created with a 500 kHz Visumax femtosecond laser (Carl Zeiss Meditec AG, Jena, Germany). The optical zone was 6.5 to 6.6 mm, the cap diameter was 7.6 mm to 7.7 mm, and the cap thickness ranged between 120 μm to 130 μm. After the blunt separation, the lenticule was extracted from a 2.0 mm incision at 120 degrees.

After the surgery, the patients were administered 0.5% levofloxacin drops (Santen Pharmaceutical Co., Ltd., Japan) and 0.5% Loteprednol Etabonate Ophthalmic Suspension (Bausch & Lomb Incorporated, United States) four times a day for 1 month. The preservative-free artificial tear was applied and adjusted according to the patient’s symptoms.

### 2.5. Patient-reported visual disturbance evaluation

The patients were required to complete the quality of vision questionnaire to assess visual disturbance 3 months postoperatively according to their situation within the last week. The questionnaire comprised eight types of visual disturbances frequently occurring following corneal refractive surgery, including glare, halo, starburst, hazy vision, double vision, vision fluctuation, focusing difficulty and difficulty in judging distance. The questionnaire assesses each visual disturbance in three dimensions: frequency (never, 0; occasionally, 1; quite often, 2; very often, 3), severity (not at all, 0; mild, 1; moderate, 2; severe, 3), and bothersome (not at all, 0; a little, 1; quite, 2; very, 3).

### 2.6. Statistical analysis

The statistical analysis was conducted using SPSS 26.0 (IBM, Armonk, United States). The graphics were generated with Microsoft Excel (2020, Microsoft Corp). The spherical equivalent (SE) was calculated as the sphere plus half of the cylinder diopter. The continuous variable was presented as mean and standard deviation, and the categorical variable was shown in number and percentage. The Shapiro–Wilk test was performed to determine the normality of the data distribution. Spearman’s correlation analysis was performed between DVA and quality of vision score for each visual disturbance.

For subgroup analysis, the patients were stratified as normal or abnormal according to the quality of vision score. “Normal” was defined as reporting “never” in the assessment of the frequency, “not at all” in severity and bothersome evaluation for a certain visual disturbance. Otherwise, it would be defined as “abnormal.” Further, the subjects were divided according to the procedure. Single factor linear model was applied to analyze the difference in DVA between normal and abnormal subgroups. *p* < 0.05 was considered statistically significant.

## 3. Results

Ninety-five patients were included in the study. The characteristic of the patients are summarized in Table 1. The average age of the included patients was 27.6 ± 6.4 years. Preoperatively, the mean spherical equivalent (SE) was −5.33 ± 1.70 D. The number of eyes enrolled undergone PRK, FS-LASIK, and SMILE was 28, 88 and 74, respectively. The preoperative characteristics of participants for different surgical procedures are summarized in Supplementary Table 1.

**Table 1:** Preoperative characteristics of the participants.

Parameters	Mean (SD)	Range (min to max)
Age (yrs)	27.6 (6.4)	18 to 40
Sphere (D)	−4.95 (1.59)	−9.25 to −1.50
Cylinder (D)	−0.74 (0.65)	−2.75 to 0
Spherical equivalent (D)	−5.33 (1.70)	−9.88 to −1.88
LogMAR CDVA	−0.07 (0.06)	−0.2 to 0.1
Central corneal thickness (μm)	546.45 (30.50)	485 to 629
Average keratometry (D)	43.20 (1.43)	38.79 to 46.82

CDVA, corrected distance visual acuity; D, diopter; SD, standard deviation.

### 3.1. Static vision and refraction

**Figure 1:**
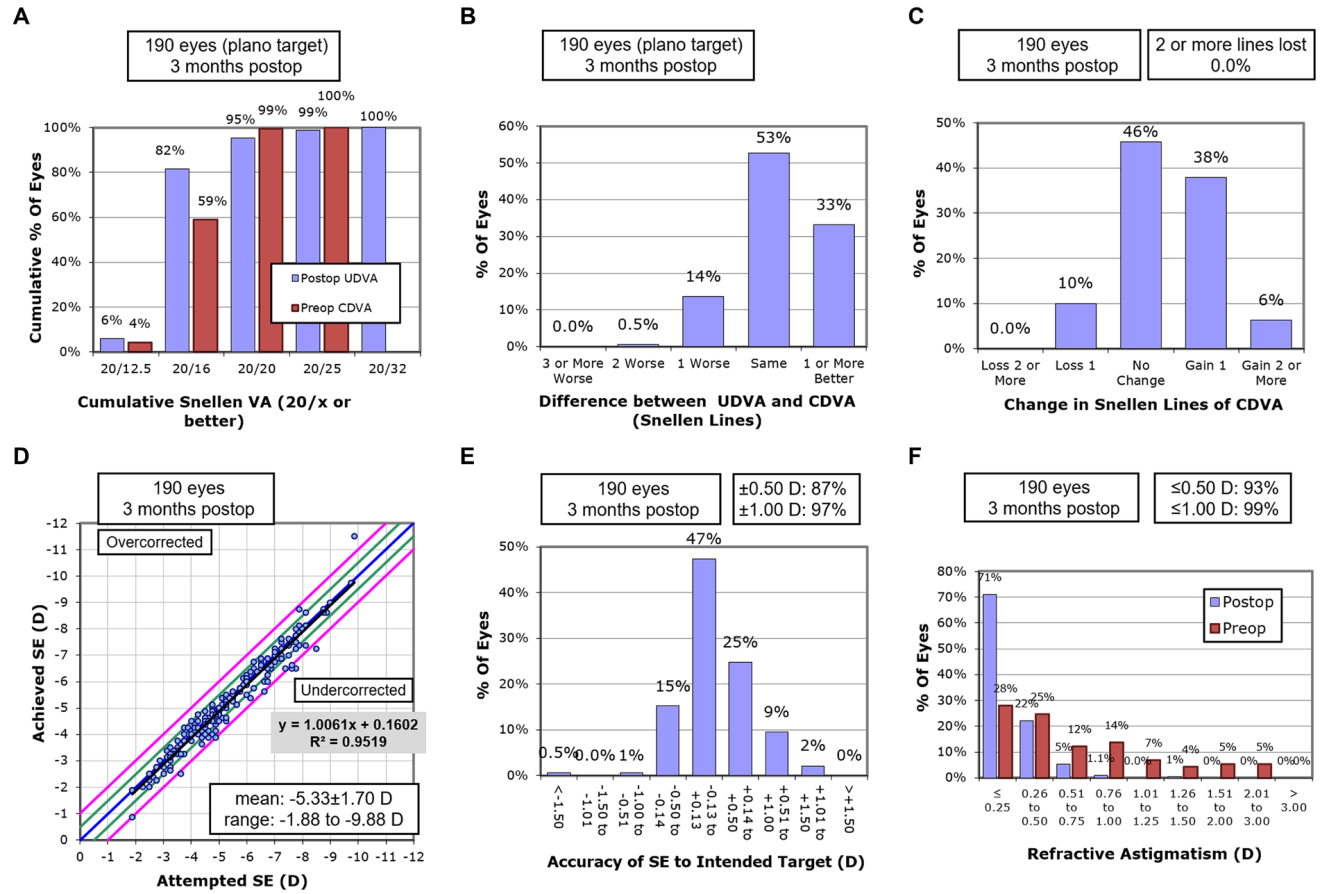
Postoperative static vision outcomes. **(A)** Cumulative visual acuity of postoperative UDVA and preoperative CDVA (*n* = 190 eyes). **(B)** Difference in the Snellen lines between postoperative UDVA and preoperative CDVA (*n* = 190 eyes). **(C)** Changes in the Snellen lines between postoperative and preoperative CDVA (*n* = 190 eyes). **(D)** Scatter diagram of attempted versus achieved SE. The oblique line separates the overcorrection and undercorrection (*n* = 190 eyes). **(E)** The accuracy of the SE to the intended target (*n* = 190 eyes). **(F)** The histogram of preoperative and postoperative refractive astigmatism (*n* = 190 eyes). (CDVA, corrected distance visual acuity; SE, spherical equivalent; UDVA, uncorrected distance visual acuity).

The result of postoperative static vision is summarized in Figure 1. The cumulative preoperative CDVA and postoperative UDVA are demonstrated in Figure 1A. The preoperative CDVA was −0.07 ± 0.06 LogMAR, and the postoperative UDVA improved to −0.08 ± 0.06 at 3 months. The preoperative UDVA in 86% of eyes was the same or better than preoperative CDVA, as shown in Figure 1B. The postoperative CDVA at 3 months was −0.10 ± 0.05. There was a significant improvement in CDVA at 3 months (*p* < 0.001), and 90% of eyes had the same or improved CDVA, as shown in Figure 1C. Figure 1D plotted the achieved SE at 3 months versus attempted SE, and a significant association was observed between achieved and attempted SE (R2 = 0.95, *p* < 0.001). Figure 1E demonstrated the accuracy of SE to the intended target. One hundred and sixty-six (87%) of the 190 eyes were within ±0.5D of attempted SE. Figure 1F demonstrated the histogram of pre-and postoperative astigmatism. The cylinder of 91% of eyes was within 0.5D at 3 months.

### 3.2. Patient-reported visual disturbance

**Figure 2:**
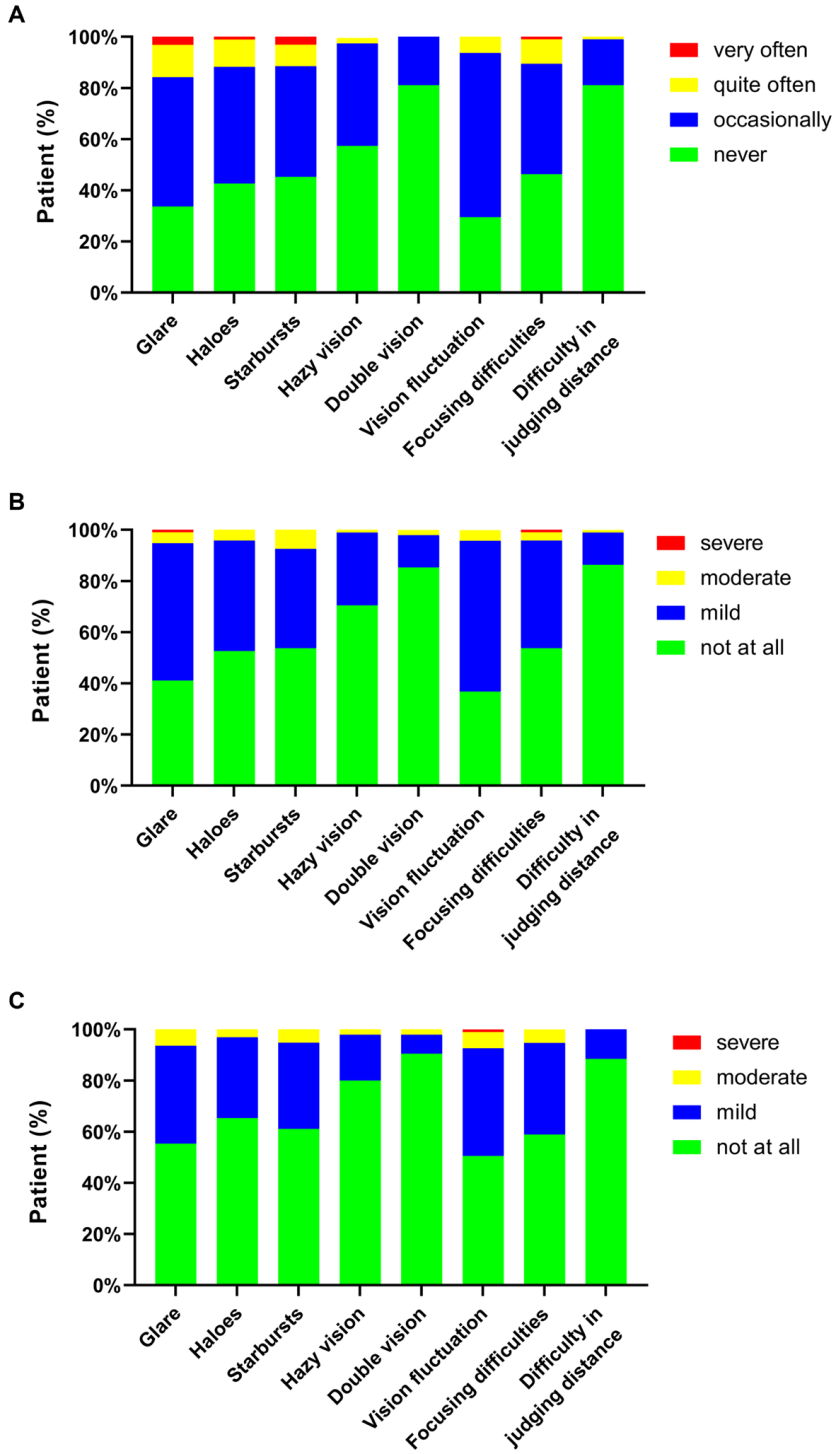
Stacked histogram for visual disturbance frequency **(A)**, severity **(B)**, and bothersome **(C)** after corneal refractive surgery. The histogram was constructed according to the percentage of patients with or without the symptoms.

The histogram of each visual disturbance is demonstrated in Figure 2. The most frequently reported visual disturbance was the fluctuation in vision (70.5%), followed by glare (66.3%) and halo (57.4%), respectively, reporting these symptoms “occasionally,” “quite often,” or “very often” (Figure 2A). The least occurred patient-reported symptoms were double vision (18.9%) and difficulty in judging distance (18.9%). Similarly, vision fluctuation was reported as the most severe and bothersome symptom, and 63.2 and 49.5% of patients reported “mild,” “moderate,” or “severe” in severity and bothersome evaluation of vision fluctuation, respectively (Figures 2B,C).

### 3.3. Correlation between postoperative DVA and visual disturbance

The postoperative DVA were 0.108 ± 0.074 and 0.146 ± 0.077 LogMAR for 40 and 80 degrees per second (dps). The total score for each visual disturbance was calculated as the sum of frequency, severity and bothersome score. The results of the correlation analysis between postoperative DVA and visual disturbance total score are demonstrated in Table 2. No visual disturbance total score was statistically associated with 40 dps postoperative DVA (*p* > 0.05 respectively). Postoperative DVA at 80 dps was significantly associated with the score of haloes (*R* = 0.214, *p* = 0.038) and difficulty in judging distance (*R* = 0.206, *p* = 0.046).

**Table 2:** Spearman correlation analysis between postoperative DVA and quality of vision total score* for each visual disturbance.

	40 dps		80 dps	
	*R*	*p* value	*R*	*p* value
Glare^*^	0.105	0.315	0.036	0.731
Haloes^*^	0.194	0.061	0.214*	**0.038**
Starbursts^*^	−0.073	0.484	−0.027	0.798
Hazy vision^*^	0.107	0.303	−0.014	0.895
Double vision^*^	−0.008	0.940	0.133	0.198
Vision fluctuation^*^	0.104	0.317	0.102	0.324
Focusing difficulties^*^	−0.009	0.933	0.091	0.380
Difficulty in judging distance^*^	0.155	0.135	0.206*	**0.046**

*Quality of vision total score for each visual disturbance was calculated as the sum of frequency, severity and bothersome score.

Boldface values indicated statistical significance at *p* < 0.05 level.

dps, degree per second; DVA, dynamic vision acuity.

The association between postoperative DVA and the visual disturbance score of each dimension is shown in Supplementary Table 2. The severity of haloes was correlated with 40 dps postoperative DVA (*R* = 0.221, *p* = 0.031) and the bothersome of haloes was correlated with 80 dps postoperative DVA (*R* = 0.243, *p* = 0.018). The postoperative DVA at 80 dps was significantly related to the frequency (*R* = 0.205, *p* = 0.046) and severity (*R* = 0.203, *p* = 0.049) of difficulty in judging distance.

### 3.4. DVA subgroup analysis by visual distance score

The patients were classified as normal or abnormal according to the score of each visual distance. The result of subgroup analysis for DVA in normal and abnormal patients was demonstrated in Table 3. Significant worse postoperative DVA at 40 (0.123 ± 0.074) was observed in patients with haloes than those without (0.088 ± 0.071, *p* = 0.024). The DVA at 80 dps for patients without haloes was 0.129 ± 0.076, which was significantly better than that with haloes (0.161 ± 0.075, *p* = 0.047). In addition, there was a significant difference in 80 dps DVA between normal (0.138 ± 0.074) and abnormal (0.181 ± 0.082) patients as for difficulty in judging distance (*p* = 0.029).

**Table 3:** DVA subgroup analysis by quality of vision total score* for each visual disturbance.

	40 dps			80 dps		
	Normal^†^	Abnormal^†^	*p* value^‡^	Normal^†^	Abnormal^†^	*p* value^‡^
	Mean ± standard deviation			Mean ± standard deviation		
Glare^*^	0.104 ± 0.071	0.111 ± 0.077	0.677	0.152 ± 0.074	0.143 ± 0.080	0.635
Haloes^*^	**0.088 ± 0.071**	**0.123 ± 0.074**	**0.024**	**0.129 ± 0.076**	**0.161 ± 0.075**	**0.047**
Starbursts^*^	0.117 ± 0.075	0.101 ± 0.074	0.314	0.152 ± 0.069	0.141 ± 0.084	0.490
Hazy vision^*^	0.107 ± 0.073	0.112 ± 0.077	0.737	0.152 ± 0.077	0.141 ± 0.077	0.522
Double vision^*^	0.108 ± 0.073	0.111 ± 0.082	0.859	0.142 ± 0.082	0.166 ± 0.050	0.230
Vision fluctuation^*^	0.103 ± 0.082	0.110 ± 0.072	0.679	0.140 ± 0.083	0.149 ± 0.075	0.641
Focusing difficulty^*^	0.108 ± 0.076	0.108 ± 0.074	0.995	0.141 ± 0.085	0.150 ± 0.071	0.562
Difficulty in judging distance^*^	0.103 ± 0.073	0.133 ± 0.078	0.124	**0.138 ± 0.074**	**0.181 ± 0.082**	**0.029**

* Quality of vision total score for each visual disturbance was calculated as the sum of frequency, severity and bothersome score.

† Normal was defined as “never” in the assessment of the frequency, and “not at all” in severity and bothersome evaluation for a certain visual disturbance. Otherwise, it would be defined as abnormal.

‡ Calculated with single factor linear model.

Boldface values indicated statistical significance at *p* < 0.05 level.

dps, degree per second; DVA, dynamic vision acuity; SD, standard deviation.

The further subgroup analysis for postoperative DVA by visual disturbance score of each dimension is demonstrated in Supplementary Table 3. The result showed a significant difference in 40 dps DVA between patients who suffered from haloes and patients without experiencing haloes (frequency, *p* = 0.033; severity, *p* = 0.034) and the result was similar for DVA at 80 dps for the assessment dimension of frequency (*p* = 0.027) and bothersome (*p* = 0.009). The patients with bothersome hazy vision had worse 40 dps DVA than those without bothersome (*p* = 0.04). Significant difference was found in 80 dps DVA between patients with and without difficulty in judging distance (*p* = 0.029).

### 3.5. DVA subgroup analysis by surgical procedure.

The patients were stratified according to the surgical procedure. The postoperative DVA in normal and abnormal patients of each visual disturbance in three surgical subgroups was summarized in Table 4. There was no significant difference in 40 or 80 dps DVA between patients with and without visual distance in PRK or SMILE subgroup. For patients who had undergone FS-LASIK, significantly worse postoperative DVA at 40 (0.133 ± 0.077) and 80 dps (0.171 ± 0.083) was observed in patients with haloes than those without haloes (40 dps, 0.078 ± 0.067, *p* = 0.026; 80 dps, 0.116 ± 0.083, *p* = 0.047). Additionally, the FS-LASIK patients without difficulty in judging distance had significantly better DVA at 40 (0.098 ± 0.078) and 80 dps (0.135 ± 0.079) than those with abnormal symptoms (40 dps, 0.179 ± 0.033, *p* = 0.003; 80 dps, 0.218 ± 0.080, *p* = 0.006).

**Table 4:** DVA subgroup analysis by surgical procedure for total score* of each visual disturbance.

	40 dps			80 dps		
	Normal^†^	Abnormal^†^	*p* value^‡^	Normal^†^	Abnormal^†^	*p* value^‡^
	Mean ± standard deviation			Mean ± standard deviation		
PRK (*n* = 14)						
Glare^*^	0.128 ± 0.099	0.088 ± 0.063	0.371	0.198 ± 0.054	0.114 ± 0.064	0.031
Haloes^*^	0.113 ± 0.057	0.090 ± 0.114	0.640	0.155 ± 0.052	0.150 ± 0.091	0.907
Starbursts^*^	0.130 ± 0.047	0.086 ± 0.088	0.324	0.190 ± 0.037	0.118 ± 0.075	0.071
Hazy vision^*^	0.103 ± 0.073	0.101 ± 0.083	0.981	0.173 ± 0.056	0.128 ± 0.078	0.283
Double vision^*^	0.115 ± 0.073	0.054 ± 0.083	0.240	0.141 ± 0.079	0.154 ± 0.051	0.790
Vision fluctuation^*^	0.100 ± 0.059	0.103 ± 0.089	0.951	0.143 ± 0.068	0.144 ± 0.078	0.964
Focusing difficulty^*^	0.163 ± 0.00	0.085 ± 0.079	0.126	0.167 ± 0.081	0.138 ± 0.072	0.555
Difficulty in judging distance^*^	0.118 ± 0.071	0.006 ± 0.009	0.053	0.146 ± 0.077	0.131 ± 0.044	0.803
FS-LASIK (*n* = 45)						
Glare^*^	0.101 ± 0.071	0.123 ± 0.082	0.368	0.149 ± 0.084	0.156 ± 0.088	0.809
Haloes^*^	**0.078 ± 0.067**	**0.133 ± 0.077**	**0.026**	**0.116 ± 0.083**	**0.171 ± 0.083**	**0.047**
Starbursts^*^	0.113 ± 0.084	0.119 ± 0.074	0.801	0.141 ± 0.077	0.164 ± 0.093	0.370
Hazy vision^*^	0.115 ± 0.076	0.119 ± 0.086	0.869	0.150 ± 0.091	0.162 ± 0.075	0.680
Double vision^*^	0.114 ± 0.078	0.121 ± 0.082	0.790	0.146 ± 0.097	0.171 ± 0.053	0.380
Vision fluctuation^*^	0.140 ± 0.092	0.110 ± 0.074	0.299	0.157 ± 0.108	0.153 ± 0.081	0.898
Focusing difficulty^*^	0.101 ± 0.083	0.131 ± 0.071	0.199	0.142 ± 0.101	0.166 ± 0.067	0.353
Difficulty in judging distance ^*^	**0.098 ± 0.078**	**0.179 ± 0.033**	**0.003**	**0.135 ± 0.079**	**0.218 ± 0.080**	**0.006**
SMILE (*n* = 36)						
Glare^*^	0.098 ± 0.063	0.104 ± 0.075	0.806	0.134 ± 0.062	0.139 ± 0.074	0.848
Haloes^*^	0.085 ± 0.081	0.116 ± 0.057	0.194	0.127 ± 0.081	0.147 ± 0.056	0.387
Starbursts^*^	0.118 ± 0.074	0.086 ± 0.064	0.170	0.155 ± 0.065	0.122 ± 0.070	0.156
Hazy vision^*^	0.095 ± 0.070	0.113 ± 0.069	0.468	0.149 ± 0.056	0.132 ± 0.080	0.468
Double vision^*^	0.100 ± 0.070	0.15^#^	0.488	0.138 ± 0.070	0.138^#^	0.996
Vision fluctuation^*^	0.079 ± 0.077	0.114 ± 0.063	0.147	0.128 ± 0.073	0.143 ± 0.067	0.521
Focusing difficulty^*^	0.108 ± 0.070	0.094 ± 0.071	0.558	0.136 ± 0.064	0.140 ± 0.075	0.882
Difficulty in judging distance^*^	0.102 ± 0.069	0.098 ± 0.081	0.896	0.138 ± 0.069	0.140 ± 0.074	0.947

* Quality of vision total score for each visual disturbance was calculated as the sum of frequency, severity and bothersome score.

† Normal was defined as “never” in the assessment of the frequency, and “not at all” in severity and bothersome evaluation for a certain visual disturbance. Otherwise, it would be defined as abnormal.

‡ Calculated with single factor linear model.

# Only one patient reported double vision in SMILE subgroup.

Boldface values indicated statistical significance at *p* < 0.05 level.

dps, degree per second; DVA, dynamic vision acuity; SD, standard deviation.

## 4. Discussion

Corneal refractive surgery is effective in correcting myopia. DVA tests and patient-reported quality of vision are methods to assess functional vision that could better reflect real-life scenarios. The present research aims to investigate the impact of commonly occurring visual disturbance on DVA in myopic patients after corneal refractive surgery. We found that patients with haloes and difficulty in judging distance have significantly worse DVA than those without visual disturbance postoperatively. To the best of our knowledge, this was the first research study to demonstrate the relationship between DVA and subjective visual disturbance in postoperative patients.

Subjective visual disturbance commonly occurs after corneal refractive surgery and is gradually alleviated in long-term observation (Schallhorn et al., 2016; Damgaard et al., 2018). In the present research, we assess eight types of visual disturbances regarding frequency, severity and bothersome in myopic patients 3 months after the surgery. The result demonstrated that the most frequently reported visual disturbance is fluctuation in vision, which occurred in 70.5% of the patients, followed by glare (66.3%). The assessment of severity and bothersome of the visual symptoms are similar. The results are largely consistent with the previous research on subjective visual symptoms; for example, Ang et al. (2015) reported fluctuation in vision as the most severe symptom 3 months after SMILE and FS-LASIK, Damgaard et al. (2018) ranked fluctuation and glare amongst the three most severe visual symptoms 3 months after SMILE and FS-LASIK, and He et al. (2022) recorded glare as one of the most commonly occurred visual symptoms 6 months after SMILE and FS-LASIK. In a long-term observation with a mean follow-up duration of 24 months, Schmelter et al. (2019) and Siedlecki et al. (2020) also demonstrated that fluctuation in vision and glare are the most commonly reported visual symptoms after SMILE. Thus, fluctuation in vision and glare appears to be essential visual disturbances during the short- and long-term postoperative period, which might be attributed to the postoperative disruption of the tear film on visual quality.

Previous research on corneal refractive surgery mainly focuses on static vision, including visual acuity, contrast sensitivity, etc. DVA is crucial in our daily life as the moving object account for most of the visual objects in daily tasks. The postoperative DVA were 0.108 ± 0.074 and 0.146 ± 0.077 LogMAR for 40 and 80 dps. The outcome was similar to previous research on low and high-speed DVA in patients after corneal refractive surgery (Wang et al., 2023a,b). We found that DVA was correlated with postoperative visual symptoms, including haloes and difficulty in judging distance. The patients with the haloes have significantly worse low and high-speed DVA than those without visual complaints. The achievement of DVA requires the coordination of complicated eye movement to track the trajectory of the moving object. The haloes worsen the visibility around the moving object. It might affect the identification and pursuit of a moving object when it moves out of the sharpest visual field. Thus, the haloes influence the visual acuity to identify the moving object.

Distance judging requires depth perception, which is the ability to coordinate the subtle differences between the images received by two eyes (Parker, 2007). The present research demonstrated that patients with difficulty judging distance have the worse high-speed DVA. However, previous research did not show a worse DVA in participants with worse depth perception in healthy participants (Kohmura et al., 2013; Chang et al., 2015). Further analysis showed a significantly positive correlation between haloes and difficulty in judging distance score (*R* = 0.352, *p* < 0.001, not shown). The result might indicate that the postoperative abnormality in depth perception might be induced by the disturbance of haloes on binocular visual function and subsequently affect the DVA.

DVA is significantly associated with the performance of our daily tasks, including sports and driving. With the advanced surgical procedure and design, patients expected an improved quality of life, in addition to spectacles independence. A series of visual disturbances commonly occur after corneal refractive surgery that might affect postoperative satisfaction and quality of life. In the present research, we found that patients with haloes and difficulty judging distance have worse DVA than patients without disturbance. The result might indicate that these visual symptoms might impact the performance of dynamic vision-related daily tasks. Patients should be remand to take caution when driving or playing sports with high-speed movement if they have the bothersome of these visual symptoms. Further analysis of the influential factor on visual disturbance facilitates the avoidance of the visual disturbance from the perspective of surgical design for the patient favoring lifestyle demanding DVA.

Certain limitations exist in the present research. First, the present study is a nonrandomized cohort research. Due to nonrandomized design, the number of patients receiving different types of procedures is unbalanced. More patients who had undergone FS-LASIK were enrolled than those with PRK and SMILE. The insignificance of the association between visual disturbance and DVA in the PRK and SMILE subgroups might be due to the small sample size. Selection bias might exist in choosing the surgery, and the result could not be accurately compared among different procedures. Second, the follow-up period is relatively short. The severity and bothersome of different visual disturbances might change gradually. Long-term observance is required in future studies. Third, only DVA with horizontally moving objects was evaluated in the present research. DVA with other moving patterns, kinetic visual acuity and motion perception might be affected by the visual disturbance in patients after corneal refractive surgery, which remains to be explored in further study. Fourth, following corneal refractive surgery, pupil diameter may affect the severity and bothersome of visual disturbance that impacts the dynamic vision. However, pupil diameter was not measured during the dynamic visual acuity examination.

This study investigates the impact of patient-reported visual disturbance on DVA in myopic patients after corneal refractive surgery. The research demonstrated that postoperative DVA at 40 and 80 dps are significantly associated with haloes and difficulty judging distance. Patients with haloes have a worse 40 dps DVA, and those with haloes or difficulty in judging distance have a worse 80 dps DVA than those without the symptoms. The present study provided the basis for postoperative guidance in daily tasks involving dynamic vision when patients have a visual disturbance.

## Data availability statement

The raw data supporting the conclusions of this article will be made available by the authors, without undue reservation.

## Ethics statement

The studies involving humans were approved by Peking University Third Hospital Ethics Committee. The studies were conducted in accordance with the local legislation and institutional requirements. The participants provided their written informed consent to participate in this study.

## Author contributions

YW: Conceptualization, Data curation, Formal analysis, Funding acquisition, Investigation, Writing – original draft. YZ: Conceptualization, Formal analysis, Investigation, Resources, Writing – review & editing. TW: Formal analysis, Investigation, Writing – review & editing. XR: Formal analysis, Investigation, Writing – review & editing. YY: Formal analysis, Investigation, Writing – review & editing. XL: Resources, Supervision, Writing – review & editing. YC: Investigation, Resources, Supervision, Writing – review & editing.
