# Corrigendum: The impact of patient-reported visual disturbance on dynamic visual acuity in myopic patients after corneal refractive surgery

**DOI:** 10.3389/fnins.2024.1444519

**Published:** 2024-06-26

**Authors:** Yuexin Wang, Yu Zhang, Tingyi Wu, Xiaotong Ren, Yifei Yuan, Xuemin Li, Yueguo Chen

**Affiliations:** ^1^Department of Ophthalmology, Peking University Third Hospital, Beijing, China; ^2^Beijing Key Laboratory of Restoration of Damaged Ocular Nerve, Beijing, China

**Keywords:** visual disturbance, dynamic visual acuity, myopia, corneal refractive surgery, visual function

In the published article, there was an error in Table 4 as published. The scientific surgical name should be “photorefractive keratectomy (PRK)” instead of “laser-assisted subepithelial keratectomy (LASEK).” The corrected Table 4 appears below.

**Table 4:** DVA subgroup analysis by surgical procedure for total score^*^ of each visual disturbance.

	**40 dps**			**80 dps**		
	**Normal** ^†^	**Abnormal** ^†^	***p*** **value**^‡^	**Normal** ^†^	**Abnormal** ^†^	***p*** **value**^‡^
	**Mean** ±**standard deviation**			**Mean** ±**standard deviation**		
**PRK (14)**						
Glare^*^	0.128 ± 0.099	0.088 ± 0.063	0.371	0.198 ± 0.054	0.114 ± 0.064	0.031
Haloes^*^	0.113 ± 0.057	0.090 ± 0.114	0.640	0.155 ± 0.052	0.150 ± 0.091	0.907
Starbursts^*^	0.130 ± 0.047	0.086 ± 0.088	0.324	0.190 ± 0.037	0.118 ± 0.075	0.071
Hazy vision^*^	0.103 ± 0.073	0.101 ± 0.083	0.981	0.173 ± 0.056	0.128 ± 0.078	0.283
Double vision^*^	0.115 ± 0.073	0.054 ± 0.083	0.240	0.141 ± 0.079	0.154 ± 0.051	0.790
Vision fluctuation^*^	0.100 ± 0.059	0.103 ± 0.089	0.951	0.143 ± 0.068	0.144 ± 0.078	0.964
Focusing difficulty^*^	0.163 ± 0.00	0.085 ± 0.079	0.126	0.167 ± 0.081	0.138 ± 0.072	0.555
Difficulty in judging distance^*^	0.118 ± 0.071	0.006 ± 0.009	0.053	0.146 ± 0.077	0.131 ± 0.044	0.803
**FS-LASIK (45)**						
Glare^*^	0.101 ± 0.071	0.123 ± 0.082	0.368	0.149 ± 0.084	0.156 ± 0.088	0.809
Haloes^*^	**0.078** **±0.067**	**0.133** **±0.077**	**0.026**	**0.116** **±0.083**	**0.171** **±0.083**	**0.047**
Starbursts^*^	0.113 ± 0.084	0.119 ± 0.074	0.801	0.141 ± 0.077	0.164 ± 0.093	0.370
Hazy vision^*^	0.115 ± 0.076	0.119 ± 0.086	0.869	0.150 ± 0.091	0.162 ± 0.075	0.680
Double vision^*^	0.114 ± 0.078	0.121 ± 0.082	0.790	0.146 ± 0.097	0.171 ± 0.053	0.380
Vision fluctuation^*^	0.140 ± 0.092	0.110 ± 0.074	0.299	0.157 ± 0.108	0.153 ± 0.081	0.898
Focusing difficulty^*^	0.101 ± 0.083	0.131 ± 0.071	0.199	0.142 ± 0.101	0.166 ± 0.067	0.353
Difficulty in judging distance ^*^	**0.098** **±0.078**	**0.179** **±0.033**	**0.003**	**0.135** **±0.079**	**0.218** **±0.080**	**0.006**
**SMILE (36)**						
Glare^*^	0.098 ± 0.063	0.104 ± 0.075	0.806	0.134 ± 0.062	0.139 ± 0.074	0.848
Haloes^*^	0.085 ± 0.081	0.116 ± 0.057	0.194	0.127 ± 0.081	0.147 ± 0.056	0.387
Starbursts^*^	0.118 ± 0.074	0.086 ± 0.064	0.170	0.155 ± 0.065	0.122 ± 0.070	0.156
Hazy vision^*^	0.095 ± 0.070	0.113 ± 0.069	0.468	0.149 ± 0.056	0.132 ± 0.080	0.468
Double vision^*^	0.100 ± 0.070	0.15^#^	0.488	0.138 ± 0.070	0.138^#^	0.996
Vision fluctuation^*^	0.079 ± 0.077	0.114 ± 0.063	0.147	0.128 ± 0.073	0.143 ± 0.067	0.521
Focusing difficulty^*^	0.108 ± 0.070	0.094 ± 0.071	0.558	0.136 ± 0.064	0.140 ± 0.075	0.882
Difficulty in judging distance ^*^	0.102 ± 0.069	0.098 ± 0.081	0.896	0.138 ± 0.069	0.140 ± 0.074	0.947

^*^Quality of vision total score for each visual disturbance was calculated as the sum of frequency, severity and bothersome score.^†^Normal was defined as “never” in the assessment of the frequency, and “not at all” in severity and bothersome evaluation for a certain visual disturbance. Otherwise, it would be defined as abnormal.^‡^Calculated with single factor linear model. ^#^Only one patient reported double vision in SMILE subgroup. Boldface values indicated statistical significance at *P* < 0.05 level. dps, degree per second; DVA, dynamic vision acuity; SD, standard deviation.

In the published article, there was also an error in the text. The scientific surgical name should be “photorefractive keratectomy (PRK)” instead of “laser-assisted subepithelial keratectomy (LASEK)”.

A correction has been made to **Abstract**, *Method*, This paragraph previously stated:

“This is a prospective nonrandomized study. Adult myopic patients receiving bilateral laser-assisted sub-epithelial keratomileusis (LASEK), femtosecond laser-assisted *in situ* keratomileusis (FS-LASIK), or small incision lenticule extraction (SMILE) with Plano target were included. Eight types of patient-reported visual disturbance were evaluated regarding frequency, severity and bothersome and dynamic visual acuity (DVA) of 40 and 80 degrees per second (dps) was measured postoperatively at 3 months.”

The corrected paragraph appears below:

“This is a prospective nonrandomized study. Adult myopic patients receiving bilateral photorefractive keratectomy (PRK), femtosecond laser-assisted *in situ* keratomileusis (FS-LASIK), or small incision lenticule extraction (SMILE) with Plano target were included. Eight types of patient-reported visual disturbance were evaluated regarding frequency, severity and bothersome and dynamic visual acuity (DVA) of 40 and 80 degrees per second (dps) was measured postoperatively at 3 months.”

A correction has been made to **Materials and methods**, *Participants*, Paragraph 2. This sentence previously stated:

“Consecutive patients undergoing bilateral laser-assisted sub-epithelial keratomileusis (LASEK), femtosecond laser-assisted *in situ* keratomileusis (FS-LASIK) or small incision lenticule extraction (SMILE) were prospectively enrolled. The inclusion criteria were as follow: (Lou et al., 2016) age 18–40 years of age (Kim et al., 2019), correction of myopia or myopic astigmatism for Plano target; (Wen et al., 2017) pre-operative and three-month postoperative corrected distance visual acuity (CDVA) 0 (LogMAR) or better. The exclusion criteria were: (Lou et al., 2016) history of severe ocular diseases, including glaucoma, retinal disease, or severe ocular surface disease; (Kim et al., 2019) vestibular dysfunction or cognitive disorder that affects the DVA test.”

The corrected paragraph appears below:

“Consecutive patients undergoing bilateral photorefractive keratectomy (PRK), femtosecond laser-assisted *in situ* keratomileusis (FS-LASIK) or small incision lenticule extraction (SMILE) were prospectively enrolled. The inclusion criteria were as follow: (Lou et al., 2016) age 18–40 years of age (Kim et al., 2019), correction of myopia or myopic astigmatism for Plano target; (Wen et al., 2017) pre-operative and three-month postoperative corrected distance visual acuity (CDVA) 0 (LogMAR) or better. The exclusion criteria were: (Lou et al., 2016) history of severe ocular diseases, including glaucoma, retinal disease, or severe ocular surface disease; (Kim et al., 2019) vestibular dysfunction or cognitive disorder that affects the DVA test.”

A correction has been made to **Materials and methods**, *Surgical procedures*, Paragraph 2. This paragraph previously stated:

“LASEK: The epithelial was removed after soaking in 20% ethanol for 20 s. The ablation was conducted with a Custom Q profile by WaveLight EX500 excimer laser (Alcon Laboratories Inc., Fort Worth, United States). After ablation, a 0.02% mitomycin C pad was covered on the residual stroma. Then the stroma was rinsed with normal saline. A bandage contact lens (Acuvue, Johnson Vision Care. Inc., United States) was placed on the cornea for protection.”

The corrected paragraph appears below:

“PRK: The epithelial was removed after soaking in 20% ethanol for 20 s. The ablation was conducted with a Custom Q profile by WaveLight EX500 excimer laser (Alcon Laboratories Inc., Fort Worth, United States). After ablation, a 0.02% mitomycin C pad was covered on the residual stroma. Then the stroma was rinsed with normal saline. A bandage contact lens (Acuvue, Johnson Vision Care. Inc., United States) was placed on the cornea for protection.”

4. A correction has been made to **Results**, Paragraph 1. This paragraph previously stated:

“Ninety-five patients were included in the study. The characteristic of the patients are summarized in Table 1. The average age of the included patients was 27.6 ± 6.4 years. Preoperatively, the mean spherical equivalent (SE) was −5.33 ± 1.70 D. The number of eyes enrolled undergone LASEK, FS-LASIK, and SMILE was 28, 88 and 74, respectively. The preoperative characteristics of participants for different surgical procedures are summarized in Supplementary Table 1.”

The corrected paragraph appears below:

“Ninety-five patients were included in the study. The characteristic of the patients are summarized in Table 1. The average age of the included patients was 27.6 ± 6.4 years. Preoperatively, the mean spherical equivalent (SE) was −5.33 ± 1.70 D. The number of eyes enrolled undergone PRK, FS-LASIK, and SMILE was 28, 88 and 74, respectively. The preoperative characteristics of participants for different surgical procedures are summarized in Supplementary Table 1.”

5. A correction has been made to **Results**, *DVA subgroup analysis by surgical procedure*, Paragraph 1. This paragraph previously stated:

“The patients were stratified according to the surgical procedure. The postoperative DVA in normal and abnormal patients of each visual disturbance in three surgical subgroups was summarized in Table 4. There was no significant difference in 40 or 80 dps DVA between patients with and without visual distance in LASEK or SMILE subgroup. For patients who had undergone FS-LASIK, significantly worse postoperative DVA at 40 (0.133 ± 0.077) and 80 dps (0.171 ± 0.083) was observed in patients with haloes than those without haloes (40 dps, 0.078 ± 0.067, *p* = 0.026; 80 dps, 0.116 ± 0.083, *p* = 0.047). Additionally, the FS-LASIK patients without difficulty in judging distance had significantly better DVA at 40 (0.098 ± 0.078) and 80 dps (0.135 ± 0.079) than those with abnormal symptoms (40 dps, 0.179 ± 0.033, *p* = 0.003; 80 dps, 0.218 ± 0.080, *p* = 0.006).”

The corrected paragraph appears below:

“The patients were stratified according to the surgical procedure. The postoperative DVA in normal and abnormal patients of each visual disturbance in three surgical subgroups was summarized in Table 4. There was no significant difference in 40 or 80 dps DVA between patients with and without visual distance in PRK or SMILE subgroup. For patients who had undergone FS-LASIK, significantly worse postoperative DVA at 40 (0.133 ± 0.077) and 80 dps (0.171 ± 0.083) was observed in patients with haloes than those without haloes (40 dps, 0.078 ± 0.067, *p* = 0.026; 80 dps, 0.116 ± 0.083, *p* = 0.047). Additionally, the FS-LASIK patients without difficulty in judging distance had significantly better DVA at 40 (0.098 ± 0.078) and 80 dps (0.135 ± 0.079) than those with abnormal symptoms (40 dps, 0.179 ± 0.033, *p* = 0.003; 80 dps, 0.218 ± 0.080, *p* = 0.006).”

A correction has been made to **Discussion**, Paragraph 6. This paragraph previously stated:

“Certain limitations exist in the present research. First, the present study is a nonrandomized cohort research. Due to nonrandomized design, the number of patients receiving different types of procedures is unbalanced. More patients who had undergone FS-LASIK were enrolled than those with LASEK and SMILE. The insignificance of the association between visual disturbance and DVA in the LASEK and SMILE subgroups might be due to the small sample size. Selection bias might exist in choosing the surgery, and the result could not be accurately compared among different procedures. Second, the follow-up period is relatively short. The severity and bothersome of different visual disturbances might change gradually. Long-term observance is required in future studies. Third, only DVA with horizontally moving objects was evaluated in the present research. DVA with other moving patterns, kinetic visual acuity and motion perception might be affected by the visual disturbance in patients after corneal refractive surgery, which remains to be explored in further study. Fourth, following corneal refractive surgery, pupil diameter may affect the severity and bothersome of visual disturbance that impacts the dynamic vision. However, pupil diameter was not measured during the dynamic visual acuity examination.”

The corrected paragraph appears below:

“Certain limitations exist in the present research. First, the present study is a nonrandomized cohort research. Due to nonrandomized design, the number of patients receiving different types of procedures is unbalanced. More patients who had undergone FS-LASIK were enrolled than those with PRK and SMILE. The insignificance of the association between visual disturbance and DVA in the PRK and SMILE subgroups might be due to the small sample size. Selection bias might exist in choosing the surgery, and the result could not be accurately compared among different procedures. Second, the follow-up period is relatively short. The severity and bothersome of different visual disturbances might change gradually. Long-term observance is required in future studies. Third, only DVA with horizontally moving objects was evaluated in the present research. DVA with other moving patterns, kinetic visual acuity and motion perception might be affected by the visual disturbance in patients after corneal refractive surgery, which remains to be explored in further study. Fourth, following corneal refractive surgery, pupil diameter may affect the severity and bothersome of visual disturbance that impacts the dynamic vision. However, pupil diameter was not measured during the dynamic visual acuity examination.”

The authors apologize for these errors and state that this does not change the scientific conclusions of the article in any way. The original article has been updated.

