# Supplementary material for: The impact of patient-reported visual disturbance on dynamic visual acuity in myopic patients after corneal refractive surgery
Source: Front Neurosci. 2023 Oct 10;17:1278626. doi: 10.3389/fnins.2023.1278626 (PMC10594990; doi:10.3389/fnins.2023.1278626)
Supplement: Supplementary file 1 [file Table_1.DOCX]

Supplementary Material

Supplementary Table 1. Preoperative characteristics of the participants for different surgical procedures.

| Parameters | LASEK | FS-LASIK | SMILE |
| --- | --- | --- | --- |
| Number (eye) | 28 | 88 | 74 |
| Age (yrs), mean (SD) | 27.9 (7.1) | 27.1 (5.8) | 28.1 (6.9) |
| Sphere (D), mean (SD) | -4.19 (0.73) | -5.74 (1.68) | -4.31 (1.26) |
| Cylinder (D), mean (SD) | -0.48 (0.44) | -1.05 (0.75) | -0.48 (0.38) |
| Spherical equivalent (D), mean (SD) | -4.43 (0.44) | -6.26 (1.74) | -4.55 (1.32) |
| LogMAR CDVA, mean (SD) | -0.07 (0.05) | -0.07 (0.06) | -0.08 (0.06) |
| Central corneal thickness (μm), mean (SD) | 524.71 (39.05) | 543.25 (22.07) | 559.17 (30.23) |
| Average keratometry (D), mean (SD) | 43.13 (1.53) | 43.37 (1.28) | 43.02 (1.57) |

Abbreviation: CDVA = corrected distance visual acuity; D = diopter; FS-LASIK = femtosecond laser-assisted in situ keratomileusis; LASEK = laser-assisted sub-epithelial keratomileusis; SD = standard deviation; SMILE = small incision lenticule extraction.

Supplementary Table 2. Spearman correlation analysis between postoperative DVA and quality of vision score for each visual disturbance

|  |  | 40 dps | | 80 dps | |
| --- | --- | --- | --- | --- | --- |
|  |  | R | P value | R | P value |
| Glare | Frequency | 0.006 | 0.952 | -0.039 | 0.707 |
|  | Severity | 0.141 | 0.172 | 0.065 | 0.534 |
|  | Bothersome | 0.190 | 0.067 | 0.109 | 0.295 |
| Haloes | Frequency | 0.166 | 0.111 | 0.171 | 0.098 |
|  | Severity | **0.221** | **0.031** | 0.177 | 0.085 |
|  | Bothersome | 0.167 | 0.106 | **0.243** | **0.018** |
| Starbursts | Frequency | -0.062 | 0.553 | -0.032 | 0.756 |
|  | Severity | -0.048 | 0.646 | 0.010 | 0.920 |
|  | Bothersome | -0.069 | 0.509 | -0.014 | 0.895 |
| Hazy vision | Frequency | 0.033 | 0.751 | -0.088 | 0.398 |
|  | Severity | 0.150 | 0.146 | 0.036 | 0.728 |
|  | Bothersome | 0.193 | 0.060 | 0.124 | 0.231 |
| Double vision | Frequency | -0.015 | 0.888 | 0.139 | 0.180 |
|  | Severity | 0.005 | 0.964 | 0.103 | 0.318 |
|  | Bothersome | 0.078 | 0.452 | 0.074 | 0.475 |
| Vision fluctuation | Frequency | 0.083 | 0.424 | 0.084 | 0.420 |
|  | Severity | 0.124 | 0.230 | 0.066 | 0.524 |
|  | Bothersome | 0.094 | 0.366 | 0.120 | 0.246 |
| Focusing difficulties | Frequency | -0.023 | 0.826 | 0.088 | 0.395 |
|  | Severity | -0.010 | 0.925 | 0.098 | 0.344 |
|  | Bothersome | 0.033 | 0.754 | 0.077 | 0.461 |
| Difficulty in judging distance | Frequency | 0.148 | 0.151 | **0.205** | **0.046** |
|  | Severity | 0.166 | 0.108 | **0.203** | **0.049** |
|  | Bothersome | 0.151 | 0.145 | 0.144 | 0.164 |

Abbreviation: dps = degree per second; DVA = dynamic vision acuity.

Boldface values indicated statistical significance at P < 0.05 level.

Supplementary Table 3. DVA subgroup analysis by quality of vision score for each visual disturbance.

|  |  | 40 dps | | | 80 dps | | |
| --- | --- | --- | --- | --- | --- | --- | --- |
|  |  | Normal^*^ | Abnormal^*^ | P value^†^ | Normal^*^ | Abnormal^*^ | P value^†^ |
|  |  | Mean ± standard deviation | |  | Mean ± standard deviation | |  |
| Glare | Frequency | 0.106±0.071 | 0.110±0.077 | 0.822 | 0.152±0.073 | 0.143±0.080 | 0.607 |
|  | Severity | 0.097±0.070 | 0.116±0.077 | 0.224 | 0.140±0.076 | 0.150±0.079 | 0.523 |
|  | Bothersome | 0.097±0.068 | 0.123±0.080 | 0.089 | 0.136±0.075 | 0.159±0.080 | 0.153 |
| Haloes | Frequency | **0.090±0.071** | **0.123±0.075** | **0.033** | **0.127±0.076** | **0.163±0.074** | **0.027** |
|  | Severity | **0.093±0.068** | **0.125±0.078** | **0.034** | 0.135±0.070 | 0.158±0.084 | 0.147 |
|  | Bothersome | 0.098±0.067 | 0.127±0.084 | 0.069 | **0.131±0.070** | **0.174±0.084** | **0.009** |
| Starbursts | Frequency | 0.114±0.077 | 0.103±0.073 | 0.481 | 0.149±0.071 | 0.144±0.083 | 0.713 |
|  | Severity | 0.114±0.073 | 0.102±0.077 | 0.446 | 0.148±0.070 | 0.145±0.086 | 0.854 |
|  | Bothersome | 0.114±0.071 | 0.099±0.080 | 0.351 | 0.147±0.068 | 0.145±0.090 | 0.900 |
| Hazy vision | Frequency | 0.107±0.073 | 0.112±0.077 | 0.737 | 0.152±0.077 | 0.141±0.077 | 0.522 |
|  | Severity | 0.101±0.072 | 0.127±0.078 | 0.118 | 0.142±0.078 | 0.156±0.075 | 0.415 |
|  | Bothersome | **0.100±0.073** | **0.139±0.075** | **0.040** | 0.140±0.079 | 0.171±0.068 | 0.118 |
| Double vision | Frequency | 0.108±0.073 | 0.111±0.082 | 0.859 | 0.142±0.082 | 0.166±0.050 | 0.230 |
|  | Severity | 0.107±0.072 | 0.114±0.089 | 0.746 | 0.143±0.080 | 0.164±0.056 | 0.345 |
|  | Bothersome | 0.106±0.072 | 0.133±0.094 | 0.291 | 0.144±0.078 | 0.163±0.070 | 0.509 |
| Vision fluctuation | Frequency | 0.106±0.081 | 0.109±0.072 | 0.835 | 0.141±0.082 | 0.148±0.076 | 0.679 |
|  | Severity | 0.103±0.079 | 0.112±0.072 | 0.565 | 0.141±0.081 | 0.149±0.076 | 0.625 |
|  | Bothersome | 0.105±0.073 | 0.112±0.076 | 0.636 | 0.137±0.078 | 0.156±0.076 | 0.243 |
| Focusing difficulties | Frequency | 0.108±0.076 | 0.108±0.074 | 0.995 | 0.141±0.085 | 0.150±0.071 | 0.562 |
|  | Severity | 0.110±0.075 | 0.106±0.074 | 0.779 | 0.143±0.085 | 0.150±0.068 | 0.657 |
|  | Bothersome | 0.107±0.074 | 0.111±0.076 | 0.804 | 0.143±0.085 | 0.151±0.066 | 0.642 |
| Difficulty in judging distance | Frequency | 0.103±0.073 | 0.133±0.078 | 0.124 | **0.138±0.074** | **0.182±0.082** | **0.029** |
|  | Severity | 0.103±0.073 | 0.141±0.075 | 0.085 | 0.140±0.075 | 0.185±0.081 | 0.053 |
|  | Bothersome | 0.104±0.073 | 0.140±0.082 | 0.136 | 0.143±0.078 | 0.169±0.667 | 0.294 |

^*^ Normal was defined as “never” in the assessment of the frequency, and “not at all” in severity and bothersome evaluation for a certain visual disturbance. Otherwise, it would be defined as abnormal.

^†^Calculated with single factor linear model.

Boldface values indicated statistical significance at P < 0.05 level.

Abbreviation: dps = degree per second; DVA = dynamic vision acuity.
